# Single Nucleotide Polymorphism (SNP)-Strings: An Alternative Method for Assessing Genetic Associations

**DOI:** 10.1371/journal.pone.0090034

**Published:** 2014-04-11

**Authors:** Douglas S. Goodin, Pouya Khankhanian

**Affiliations:** 1 Department of Neurology, University of California San Francisco, San Francisco, California, United States of America; 2 UCSF Multiple Sclerosis Center, University of California San Francisco, San Francisco, California, United States of America; Cincinnati Children’s Hospital Medical center, United States of America

## Abstract

**Background:**

Genome-wide association studies (GWAS) identify disease-associations for single-nucleotide-polymorphisms (SNPs) from scattered genomic-locations. However, SNPs frequently reside on several different SNP-haplotypes, only some of which may be disease-associated. This circumstance lowers the observed odds-ratio for disease-association.

**Methodology/Principal Findings:**

Here we develop a method to identify the two SNP-haplotypes, which combine to produce each person’s SNP-genotype over specified chromosomal segments. Two multiple sclerosis (MS)-associated genetic regions were modeled; DRB1 (a Class II molecule of the major histocompatibility complex) and MMEL1 (an endopeptidase that degrades both neuropeptides and β-amyloid). For each locus, we considered sets of eleven adjacent SNPs, surrounding the putative disease-associated gene and spanning ∼200 kb of DNA. The SNP-information was converted into an ordered-set of eleven-numbers (subject-vectors) based on whether a person had zero, one, or two copies of particular SNP-variant at each sequential SNP-location. SNP-strings were defined as those ordered-combinations of eleven-numbers (0 or 1), representing a haplotype, two of which combined to form the observed subject-vector. Subject-vectors were resolved using probabilistic methods. In both regions, only a small number of SNP-strings were present. We compared our method to the SHAPEIT-2 phasing-algorithm. When the SNP-information spanning 200 kb was used, SHAPEIT-2 was inaccurate. When the SHAPEIT-2 window was increased to 2,000 kb, the concordance between the two methods, in both of these eleven-SNP regions, was over 99%, suggesting that, in these regions, both methods were quite accurate. Nevertheless, correspondence was not uniformly high over the entire DNA-span but, rather, was characterized by alternating peaks and valleys of concordance. Moreover, in the valleys of poor-correspondence, SHAPEIT-2 was also inconsistent with itself, suggesting that the SNP-string method is more accurate across the entire region.

**Conclusions/Significance:**

Accurate haplotype identification will enhance the detection of genetic-associations. The SNP-string method provides a simple means to accomplish this and can be extended to cover larger genomic regions, thereby improving a GWAS’s power, even for those published previously.

## Introduction

Multiple sclerosis (MS) has a complex etiological basis, which involves both the genetic makeup of an individual and their environmental experiences [1]–[8]. With regard to the importance of genetics, it is notable that the life-time risk of disease for an individual from northern Europe or Canada is about 0.1% [2]. The risk in individuals who have an affected family member increases in rough proportion to the amount of shared genetic information between the affected relative and the individual [2]–[8]. Thus, third degree relatives (12.5% genetic similarity) such as first cousins, have a risk less than 1%; second degree relatives (25% genetic similarity) such as aunts and uncles have a risk of about 1–2% and first degree relatives (50% genetic similarity) such as siblings, parents, and children of an MS proband have a risk of approximately 2–5%. By contrast, in monozygotic-twins of an MS proband (100% genetic similarity) the risk increases to about 25–30% [2]–[8]. From this, it is clear that although environmental events are important for MS pathogenesis, genetic susceptibility is critical. Indeed, because genetic susceptibility seems necessary for MS to develop [9], because the necessary environmental factors seem to be “population-wide” exposures [9], and because only about 2% of the population is genetically susceptible to getting MS [9], genetics is, by far, the greatest single determinant of disease. In addition, this genetic susceptibility to multiple sclerosis (MS) seems to involve multiple genetic loci [10]–[16]. This fact has become particularly apparent from the very large genome-wide associations studies (GWAS) that have been recently published [14], [15], [17]–[21].

The largest of these was a multicenter, multi-country GWAS involving tens of thousands of cases and controls [14], [15], which identified single-nucleotide polymorphisms (SNPs) in about 100 genomic regions that were MS-associated. With notable exception of some SNPs near the DRB1 locus on chromosome 6, however, the odds ratios (OR) for almost all of these associations was quite low – i.e., mostly between OR = 1.1 and OR = 1.2 [14], [15]. This could be due to these other genetic factors having a smaller impact on MS-susceptibility compared to the DRB1 locus [14], [15]. However, it could also be due to the same SNP being present on more than one haplotype at a particular locus. If so, this may reduce substantially the measured OR for the association of a particular genomic region with the disease [11].

As a result, several haplotype-based approaches have been explored and are thought to improve the statistical power for detecting genetic associations, especially for rare causal alleles [22]. One early approach was a simple algorithm, which identified individuals who had unambiguous haplotypes of DNA sequences because they were either homozygous for every nucleotide in the sequence or because they were heterozygous at only a single site [23], [24]. For unresolved individuals, the algorithm then attempted to pair the known haplotypes with “novel” haplotypes to produce the individual’s likely genotype. If such a pair was possible, the new “novel” haplotype was presumed to be present in the population. Despite the simplicity of this method, however, probabilistic approaches (using maximum likelihood estimation or Bayesian analysis) have become the preferred method for identifying likely haplotypes [25]–[28]. In part, this has been due to expressed concerns that the simple algorithm yields haplotypes, which depend upon the order of data entry [25]–[27], that it depends upon the presence of homozygotes or single site heterozygotes to get started [24]–[27], that it identifies only the minimum number of haplotypes [26], [27], that it is more sensitive to departures from Hardy-Weinberg equilibrium (HWE) than other methods [27], and that it results in more errors compared to probabilistic approaches [27].

Nevertheless, the simplicity of the approach is notable and it seems likely that refinements in the method might overcome many of these concerns. It is the purpose of this paper to explore the utility of using an alternative analysis method to define the haplotypes that are present at a particular genetic locus within the population and to test the ability of such specific haplotypes to detect disease-associations.

## Materials and Methods

### Study Participants

The study cohort has been described in detail previously [21]. Briefly, the cohort was assembled as a prospective multicenter effort, which began in 2003. Three MS centers participated both in patient recruitment and in the collection of biological specimens. Two of the centers (Vrije Universiteit Medical Center, Amsterdam; and University Hospital Basel) were in Europe and one (University of California, San Francisco) was in the United States (US). This study consisted primarily of patients with a northern-European ancestry. Although all clinical MS-subtypes were included, most had a relapsing-remitting (RR) onset. The diagnosis of RRMS (or other subtypes of MS) was made utilizing internationally recognized criteria [29], [30]. All participating centers used identical inclusion and diagnostic criteria. Control subjects were matched with cases by age and gender. The Committee on Human Research at each of the participating centers approved the protocol and informed consent was obtained from each study participant.

### Genotyping and Quality Control

The genotyping and quality control methods utilized for the analysis of this cohort have been previously described in detail [21]. Briefly, genotyping was done at the Illumina facilities using the Sentrix HumanHap550 BeadChip. This analysis resulted in genotype information about 551,642 SNPs in 975 cases and 882 controls. DRB1*1501 genotyping was performed using a validated gene-specific TaqMan assay [21] and was only undertaken for participants from the US.

### Genetic Loci

Although several of the MS-related genetic loci were screened preliminarily (including an intergenic region), two loci were selected for detailed analysis, both of which had been previously associated with MS in a large GWAS [14]. The first locus was the DRB1 locus on Chromosome 6, for which the susceptibility allele (DRB1*1501) is known [19], [31]–[33]. DRB1 encodes a protein (a Class II molecule of the major histocompatibility complex), which binds foreign peptides derived from extracellular proteins for presentation to thymic derived lymphocytes (T-cells). It is expressed on the surface of antigen presenting cells such as dendritic cells, bone marrow derived lymphocytes (B-cells) and macrophages. This locus was picked because, for the US population, both the DRB1*1501 status and the SNP-information was available. The other locus (MMEL1) on Chromosome 1 was chosen because it was the first non-DRB1 MS-associated gene listed by the International Consortium [14]. MMEL1 is a member of the membrane (M13) metallo-endopeptidase family and is involved in the degradation of both neuropeptides and β-amyloid [34]. As it might potentially relate to MS risk, however, the function of this protein is not well defined.

### Statistical Methods

**Figure 1:**
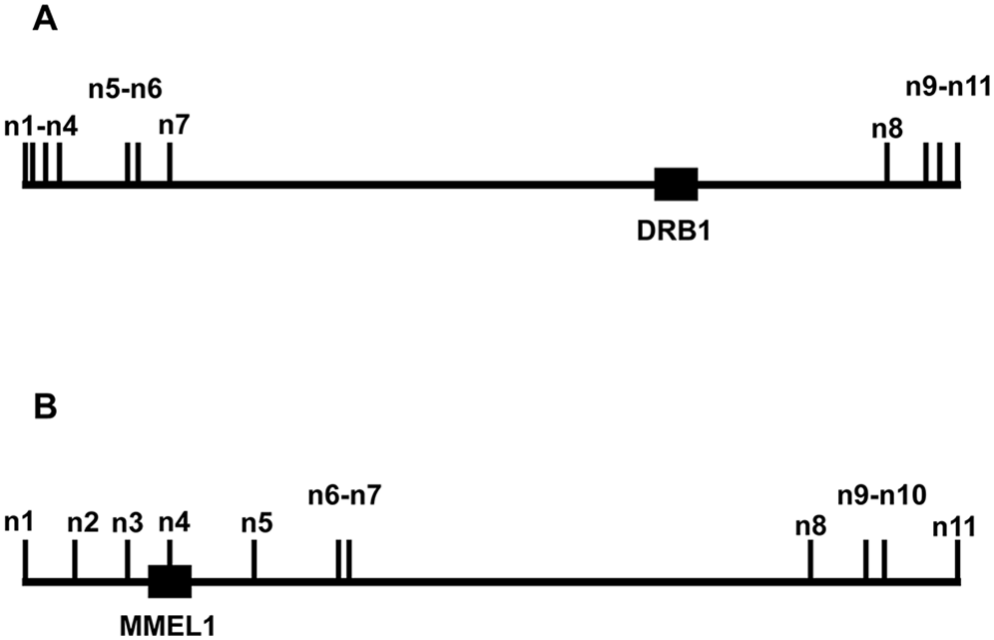
The positioning of the different SNPs used for the SNP-string analysis relative to the putative gene of interest for the DRB1 locus (A) and the MMEL1 locus (B).

To simplify program development, only eleven SNPs were used from each genomic region. The choice of eleven SNPs was arbitrary. Preliminary exploration demonstrated that the method could define haplotypes using anywhere between 3 and 24 SNPs although, in theory, there is no upper limit to the method as long as there are a sufficient number of homozygotes and single-site heterozygotes in the population. Nevertheless, for the purpose of this study, the eleven SNPs were chosen because they flanked both the most significantly associated SNP and the putative gene of interest (Figure 1). Each SNP was labeled sequentially from (n1) to (n11) based on its chromosomal location (Table 1; Figure 1). The eleven SNPs, which were analyzed at the DRB1 locus, did not include the four tagging SNPs (rs3129934, rs9267992, rs9271366, and rs3129860) identified previously [21] because these particular SNPs were not available in this dataset. Moreover, the MMEL1 region was not identified as MS-associated by this earlier study [21].

**Table 1:** SNPs used for the SNP-String Analysis.

Gene	Label	SNP‡	Chromosome	Location	Distance*
**DRB1**					
	n1	rs2239804_(G)	6	32519501	−63315.36
	n2	rs7192_(T)	6	32519624	−63192.36
	n3	rs2395182_(G)	6	32521295	−61521.36
	n4	rs3129890_(C)	6	32522251	−60565.36
	n5	rs9268832_(T)	6	32535767	−47049.36
	n6	rs6903608_(C)	6	32536263	−46553.36
	n7	rs2395185_(T)	6	32541145	−41671.36
	n8	rs477515_(T)	6	32677669	94852.64
	n9	rs2516049_(G)	6	32678378	95561.64
	n10	rs556025_(A)	6	32678858	96041.64
	n11	rs2858870_(G)	6	32680229	97412.64
**MMEL1**					
	n1	rs2234167_(A)	1	2522390	−99877.36
	n2	rs6667605_(T)	1	2534942	−87325.36
	n3	rs734999_(T)	1	2545378	−76889.36
	n4	rs3748816_(C)	1	2558908	−63359.36
	n5	rs12138909_(T)	1	2570900	−51367.36
	n6	rs11590198_(A)	1	2584062	−38205.36
	n7	rs3890745_(G)	1	2585786	−36481.36
	n8	rs4648499_(A)	1	2722807	100539.64
	n9	rs4648356_(A)	1	2732322	110054.64
	n10	rs2377041_(A)	1	2736485	114217.64
	n11	rs10909880_(C)	1	2750961	128693.64

‡ Nucleotide base (of the pair at each SNP), which is coded as having 0, 1, or 2 copies, is shown in parentheses.

*Distance from the center of each SNP-cluster. The DRB1cluster spans 160.7 kb and includes a gap of 136.5 kb between SNPs (n7) and (n8). The MMEL1 cluster spans 228.6 kb and includes a gap of 137.0 kb between SNPs (n7) and (n8).

The DRB1 cluster spans a length of 160.7 kilobases (kb) of DNA and the MMEL1 cluster spans 228.6 kb (Table 1). Of note, both clusters have a large gap in SNP-coverage of approximately 137 kb between SNPs (n7) and (n8). For each SNP-cluster, the SNP-information for each individual was converted into an ordered set (the subject-vector) of eleven ternary numbers (0, 1, or 2) based on whether they had zero, one or two copies of a particular SNP-variant for each sequential SNP in the cluster (these SNP-variants were designated according to the number of copies of the minor allele - in the control population - at each location). For example, the 5^th^ subject in the database had the DRB1 subject-vector of (20001111110), which indicated that he possessed 2 copies of (n1), 0 copies of (n2), 0 copies of (n3), and so forth. Because, essentially, all SNPs in the genome are binary, these subject-vectors are composed of two haplotypes, which either do or don’t have a particular SNP variant at each of the SNP locations. For the purpose of the present analysis, SNP-strings were vectors, defined as those specific ordered sets of eleven binary numbers (0 or 1), representing the two haplotypes (over the entire cluster span), which combined (added) to produce each observed subject-vector.

**Figure 2:**
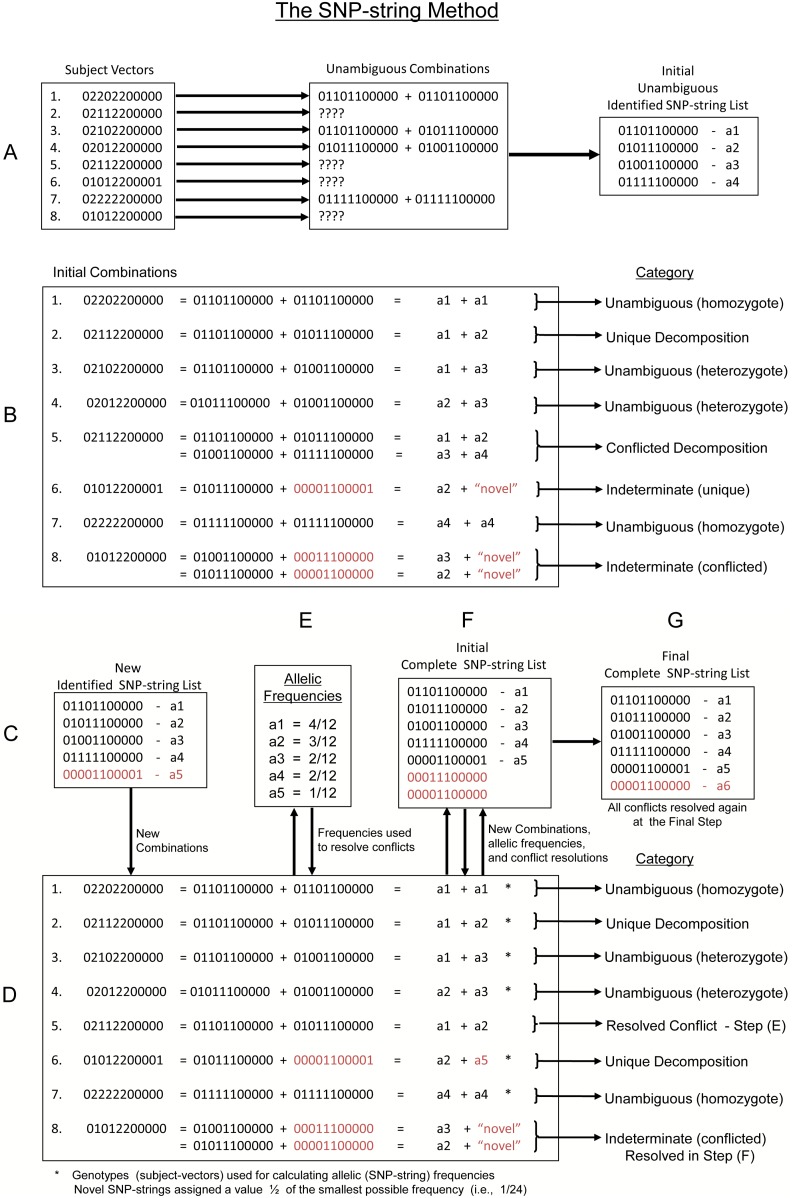
Graphical depiction of the SNP-string method presented here (see text). Subject-vectors (strings of 0s, 1s, and 2s) are searched for homozygous and single-site heterozygus individuals (A). These “unambiguous” combinations are decomposed into their constituent “unambiguous” SNP-string combinations and then a list of all the unambiguous SNP-strings that have been “identified” is compiled (A). Following this, the entire subject-vector list is decomposed into the possible combination categories (B). The “indeterminate (unique)” decompositions are used to “identify” additional SNP-strings, which are then added to the “identified” SNP-string list (B and C). The entire decomposition is then repeated until either no further SNP-strings can be added to the “identified” list or more than 4 decompositions have taken place. The final list are considered “identified” SNP-strings (C and D). Unique decompositions (of “identified” SNP- strings) and unambiguous combinations are used to calculate the allellic frequencies used for resolving the “conflicted” decompositions (E). Following this, a list of all possible addtional “novel” SNP-strings is compiled from the “indeterminate (conflicted)” decompositions (F). This list is then combined with the “identified” list to make the “complete” SNP-string list (F) and the decomposition repeated. By definition, using this “complete” list, every subject-vector will be either an unambiguous combination or a unique or conflicted decomposition. Allellic frequencies are recalculated from the unique decompositions and unambiguous combinations and these frequencies used to resolve the conflicted decompositions. Those SNP-strings that never selected (i.e., have a zero final frequency) are then dropped from the “complete” list and the decomposition repeated until all SNP-strings on the “complete” list have a non-zero final frequency (G). In the final step, persons with uncommon alleles (selected in less than 9 individuals) are then reassessed for alternative possible combinaitions with “novel” SNP-strings, these novel SNP-strings are added to the “complete” list, and the process described above is again repeated until all SNP-strings on the “complete” list have a non-zero final frequency and the SNP-string composition of every subject-vector has been selected.

**Figure 3:**
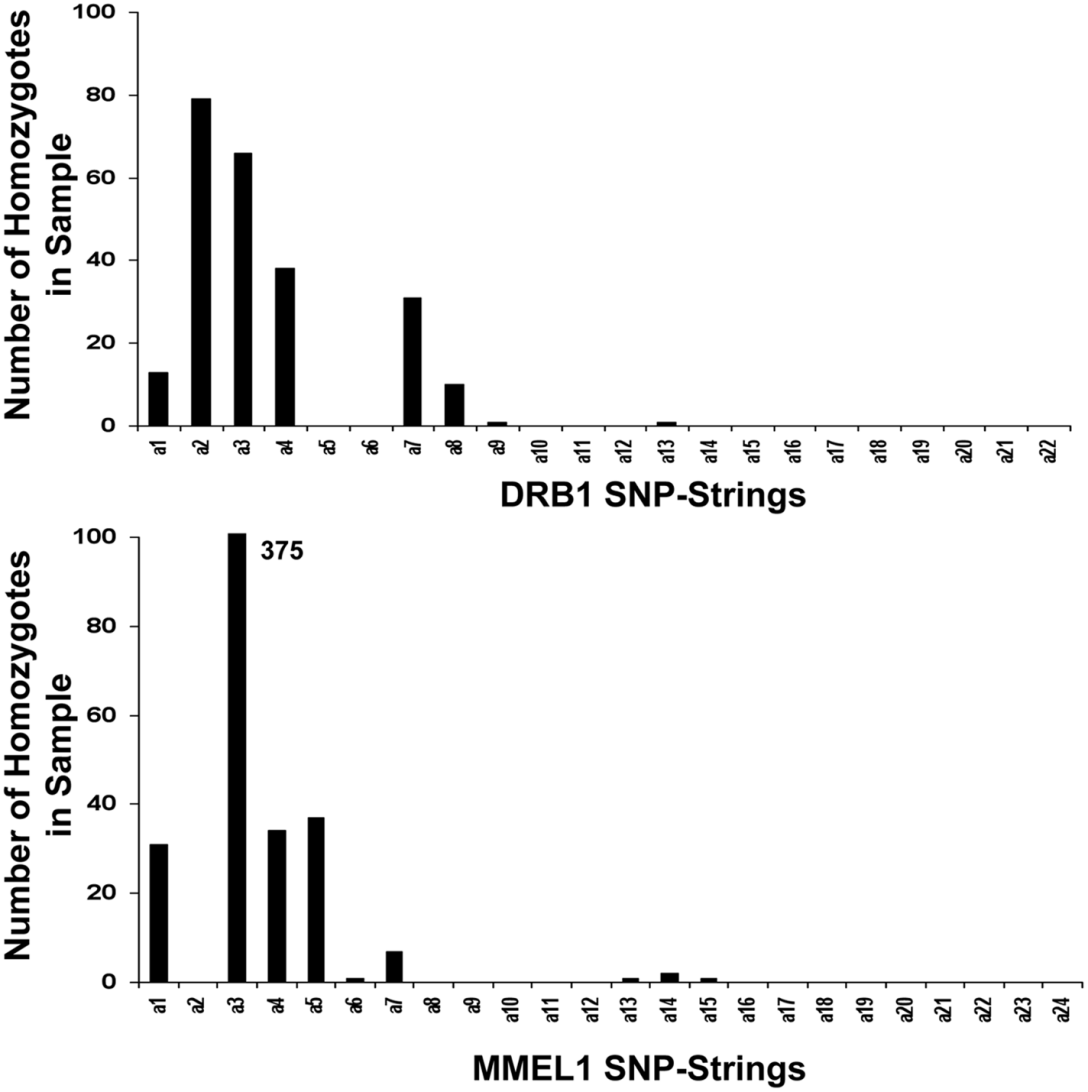
Number of homozygous SNP-strings in the DRB1 and MMEL1 clusters. NB: In the panel depicting the MMEL1 SNP-strings, the number of (a3) homozygotes was 375. The (a3) bar has been cut off at 100 in order to better illustrate the remainder of the distribution.

As with Clark’s method [23], [24], those SNP-strings that were unambiguously present in the population were identified in two ways. The first was to identify all subject-vectors, which consisted entirely of zeros (0s) and twos (2s). These individuals must be homozygous for the same SNP-string. For example, the 6^th^ subject in the database had a DRB1 subject-vector of (02202200000), which indicated that she possessed two copies of the (01101100000) SNP-string. The second method was to identify all individuals who were single SNP-heterozygotes (i.e., had subject-vectors consisting of all 0s and 2s except for having a 1 at a single SNP location). These individuals must have identical SNP-strings except for the single location where one SNP-string had a 0 and the other had a 1. For example, the 147^th^ person in the database had a DRB1 subject-vector of (20000022221), which could only arise from the combination of the SNP-strings (10000011110) and (10000011111). In this manner, a list of most common SNP-strings in the population was compiled (Figure 2). Moreover, the relative frequency of the homozygous representation of each SNP-string in the cases and controls provides an estimate of the underlying SNP-string frequency in each population (Figure 3).

Once the list of these unambiguous “identified” SNP-strings was compiled, the observed subject-vectors were decomposed into those combinations of the unambiguous “identified” SNP-strings, which could (potentially) have produced the observed subject-vector. For each person, one of three outcomes was possible. First, it might be the case that there was only one (unique) combination of two unambiguous “identified” SNP-strings, which could give rise to the observed subject-vector (Figure 2). Second, it might be that there was more than one combination of two unambiguous “identified” SNP-strings, each of which could give rise to the same observed subject-vector. In this second case, the decomposition of the observed subject-vector was said to be “conflicted” (Figure 2). And third, it might be that there was no combination of unambiguous “identified” SNP-strings, which could give rise to the observed subject-vector. In this last case, an additional “novel” (not unambiguously identified) SNP-string (or strings) must be present in the population.

Because all such “novel” SNP-strings are (presumably) low in frequency compared to the unambiguous “identified” SNP-strings, it is very likely that, when they occur, they will be heterozygous with one of the unambiguous “identified” SNP-strings. In many cases, two or more “novel” SNP-strings could, potentially, have combined with an unambiguous “identified” SNP-string to produce the same observed subject-vector. In these cases, the “novel” SNP-string, which actually underlies the observed subject-vector was said to be in an “indeterminate” (conflicted) combination (Figure 2). In these circumstances, every such “legitimate” haplotype (i.e., one which consisted of a string of zeros and ones) was added to the growing list “possible” alternative haplotypes. The SNP-strings underlying a subject-vector could also be “indeterminate” if the observed subject-vector could only be formed by the combination of two “novel” SNP-strings. In some cases, however, only a single possible “novel” SNP-string could be combined with an unambiguous “identified” SNP-string to produce the observed subject-vector (Figure 2). These were referred to as “indeterminate” (unique) combinations (Figure 2). In these cases, it is very likely that the “novel” SNP-string (so identified) actually occurs in the population. Therefore, after searching each “indeterminate” subject-vector for these unique “novel” SNP-strings, this set of newly-discovered “novel” haplotypes was added to the set of unambiguous “identified” SNP-strings to form a new list of “identified” SNP-strings. Then the entire set of observed subject-vectors was again decomposed using this new “identified” SNP-string list. After compiling this new “identified” SNP-string list, the decomposition of the subject-vectors into their constituent SNP-strings was repeated. As expected, compared to the first decomposition using only unambiguous “identified” SNP-strings, this decomposition resulted in a greater number of conflicts being found.

Additional rounds of the same procedure were repeated until no further SNP-strings could be added to the “identified” SNP-string list or until more than 4 iterations were performed. Generally, however, this process was complete after 2 or 3 cycles. After screening ∼25,000 subject-vectors using this procedure, only one individual had a genotype (subject-vector) which could not be decomposed into a pair of haplotypes, in which at least one of the pair was an “identified” SNP-string. This single individual, therefore, must either have had a heterozygous state with two unidentified “novel” SNP-strings or an error was made in their genotyping.

For the present analysis, whenever the SNP-string identification was conflicted, these conflicts were resolved based on the relative probabilities of the different allelic combinations in the population. These probabilities, in turn, were estimated from the SNP-string frequencies for all non-conflicted identifications (Figure 2; Table 2). For example, the 7^th^ person in the database had a DRB1 subject-vector of (11111011110). This could have arisen from the combination of either the (a7–a8) or the (a13–a20) SNP-strings. The probability of the latter combination (determined from the non-conflicted identifications in the whole population), however, involves two very rare SNP-strings compared to the former (Table 2) and, in this case, there is 50-fold difference in likelihood. Consequently, this particular conflict was resolved in favor of the (a7–a8) combination. In rare cases, there was little difference in likelihood between possible haplotype pairs although, more commonly, the likelihoods differed by an order of magnitude (or more) between pairs. Therefore, for the purposes of the present analysis method, all conflicts were always resolved in favor of the most likely SNP-string combination.

**Table 2:** Estimated SNP-string frequencies using either the non-conflicted or final identifications*.

	“Identified” SNP-String Frequencies (%) *																							
	a1	a2	a3	a4	a5	a6	a7	a8	a9	a10	a11	a12	a13	a14	a15	a16	a17	a18	a19	a20	a21	a22	a23	a24
**Non-Conflicted**																								
**DRB1**																								
**Cases**	8	31	13	8	1	2	12	5	2	0	1	1	3	0	0	0	0	0	2	1	7	3		
**Controls**	10	13	22	10	1	2	16	6	2	0	1	0	2	0	0	0	0	0	1	1	9	2		
**MMEL1**																								
**Cases**	11	2	46	13	13	2	4	1	1	0	0	0	1	1	1	0	0	0	1	0	0	0	0	0
**Controls**	13	3	42	13	13	2	3	2	1	0	0	0	1	1	2	0	0	0	2	0	0	0	0	0
**Final Frequency**																								
**DRB1**																								
**Cases**	8	28	16	15	1	1	12	6	2	0	1	0	2	0	0	0	0	0	1	1	5	2		
**Controls**	13	12	20	15	1	2	16	7	2	0	1	0	1	0	0	0	0	0	1	1	7	2		
**MMEL1**																								
**Cases**	13	1	46	12	13	2	5	1	1	0	0	0	1	1	1	0	0	0	1	0	0	0	0	0
**Controls**	14	1	43	11	13	2	5	1	2	0	0	0	1	1	1	0	0	0	1	0	0	0	0	0

*Percentages are rounded to the nearest percent. In every case, the number 0 does not actually represent zero because every SNP-string was possessed by, at least, one individual. “Non-Conflicted” peercents are taken from those identifications, which were not conflicted (see text). “Final” percentages are from all subjects following the SNP-string analysis. Only so-called “identified” SNP-strings are displayed in the Table but numbers are derived from the “complete” analysis (see text). All undisplayed SNP-strings on the “complete list” had frequencies, which rounded to zero.

We also generated a so-called “complete” SNP-string list. To do this we combined the lists of “possible” and “identified” SNP-strings, which had been compiled during the above analyses. We then decomposed the subject-vectors using the entire combined list. Again conflicts were resolved using the product of the estimated frequencies derived from the non-conflicted SNP-string identifications. For the “novel” SNP-strings, we assigned each the nominal estimated frequency of half the smallest possible frequency for an “identified” SNP-string (i.e., half of one divided by the number of unambiguous or non-conflicted SNP-strings in the population). Following the development of this “complete” list, the decomposition was repeated. Again, if a particular SNP-string was never observed in a non-conflicted combination, the estimated frequency was taken half the smallest possible frequency. However, for every SNP-string that was observed in a non-conflicted combination, its estimated frequency was taken as that, which had actually been observed. Using this “complete” list, 100% of the subject-vectors could be resolved into haplotypes. Following this “complete” analysis, those “novel” SNP-strings, which accounted for more than 1% of the non-conflicted identifications, were added to the final “identified” SNP-string list. Also, using this “complete” list, the decomposition was redone iteratively, eliminating those haplotypes that were never selected (i.e., which had a zero-frequency in both cases or controls), until all remaining haplotypes on the “complete” list had a non-zero final frequency and 100% of the subject-vectors could be decomposed into their constituent haplotypes (Figure 2).

Following, this penultimate decomposition, we reassessed those individuals who carried haplotypes that were found in fewer than 9 individuals. For these individuals, we created a new “possible” haplotype list, consisting of all of those “legitimate” alternative haplotypes, which could have combined with an “identified” haplotype to form the observed subject-vector and, thus, could have substituted for the very rare haplotype, which had been selected by the above procedure. The number nine was chosen because, for haplotypes having this expected number of observations (or more), there is greater than a 99.9% chance (Poisson distribution) that, at least, one example would have been found in the data. Also, the inclusion of a greater number of haplotypes on the “possible” list only serves to make the inadvertent exclusion of a rare (but present) haplotype less likely. The new list of “possible” haplotypes was added to the final “complete” list and, once again, the decomposition was redone iteratively, eliminating those haplotypes that were never selected, until all remaining haplotypes on the final “complete” list had a non-zero final frequency and 100% of the subject-vectors were decomposed. For both the DRB1 and MMEL1 loci, this last iterative decomposition made no difference to the final “complete” SNP-string list. The final SNP-string frequencies (Table 3) were calculated following this last “complete” decomposition and following the final selection process.

**Table 3:** SNP-string frequency in European and US populations*.

	Final “Identified” SNP-String Frequencies (%) *																							
	a1	a2	a3	a4	a5	a6	a7	a8	a9	a10	a11	a12	a13	a14	a15	a16	a17	a18	a19	a20	a21	a22	a23	a24
**DRB1**																								
**Cases**																								
**US**	7	27	16	13	1	1	12	6	2	1	1	0	1	0	0	0	0	0	2	0	5	1		
**EU**	8	29	15	16	1	1	11	5	2	0	1	0	2	0	0	0	0	0	0	1	4	2		
**Controls**																								
**US**	12	11	19	12	1	2	18	8	3	0	1	1	1	0	0	0	0	0	1	0	7	2		
**EU**	13	13	21	17	0	1	13	7	1	0	1	0	1	0	0	0	0	0	0	1	6	1		
**MMEL1**																								
**Cases**																								
**US**	14	1	48	12	12	2	5	1	1	1	0	1	1	0	1	0	0	0	0	0	0	0	0	0
**EU**	12	1	45	13	14	2	5	1	2	0	0	0	2	1	1	0	0	0	1	0	0	0	0	0
**Controls**																								
**US**	15	1	41	13	13	2	5	1	2	0	1	0	1	2	1	0	0	0	1	0	0	0	0	0
**EU**	14	1	45	10	13	2	5	1	1	0	0	0	1	1	1	0	0	0	2	1	0	0	0	0

*Percentages are rounded to the nearest percent. In almost every case, the number 0 does not actually represent zero. Only “identified” SNP-strings are displayed in the Table but numbers are derived from the “complete” analysis (see text).

We compared our method of phasing to the commonly-used SHAPEIT-2 method, which has been validated on several large datasets [35]. The details of this method are described elsewhere [35], [36], [37]. Briefly, the SHAPEIT-2 method combines features of SHAPEIT [36] and Impute2 [37] to enhance performance. SHAPEIT uses a Markov model to separate the haplotype-space for a given individual [36] from the set of all possible haplotype pairs consistent with a person’s genotype. Transition probabilities are estimated in local windows of a given size with a “surrogate family” approach used to select the set of templates with the smallest Hamming distances because those at short distances presumably share recent ancestry with the individual under consideration [37]. The SHAPEIT-2 method has been shown to be superior to several other methods based on its performance using several large-sample, whole-chromosome data sets from a range of SNP genotyping chips [35].

For the purposes of our analysis, we compared the haplotype predictions using the two phasing methods. The actual haplotype frequencies in the population were estimated in three manners. The first (Figure 3), was to determine the most frequent haplotypes based on the number of homozygotes in the sample population. Because both the case and control populations are at HWE, at least with respect to the DRB1 locus (9), the different haplotype frequencies can be estimated as the square root of the homozygotic frequencies [9], [38]. This method is independent of which phasing method is used. The second method was to use the haplotype frequencies, estimated from all non-conflicted haplotypes found using the phasing method presented in this study (Tables 2 and 3). These frequencies were estimated jointly, combining cases with controls, although they are presented separately in Table 2. The third method was to estimate haplotype frequencies as predicted by the SHAPEIT-2 output. All three of these methods provided substantially similar estimates for the relative frequencies of the most common haplotypes (Figure 3; Tables 2 and 3). We analyzed discrepancies between SHAPEIT-2 and our phasing algorithm by using these predicted allele frequencies to estimate the likelihood of individual phasing predictions (defined as the product of frequencies of the two phased haplotypes identified) by each algorithm.

Subjects were then grouped into homozygous carriers, heterozygous carriers and non-carriers of a particular SNP-string and tested for differences in the SNP-string distribution between patients and controls. ORs were calculated separately from homozygous and heterozygous frequencies (with non-carrier frequencies as reference) and the significance of any distribution shift assessed by a Chi Square test with 1 degree of freedom. These were compared to similar calculations for the SNPs considered individually and, in the case of the DRB1 locus, to the same calculations for the actual distribution of the DRB1*1501 allele. Because the European and American data were acquired independently and in different geographic regions but were otherwise similar, these two data sets were used separately to assess the replicability of any findings. Only the American data contained both the SNP-status and the DRB1*1501 status so that only this data could be used to correlate SNP-haplotypes with a known susceptibility genotype.

## Results

The results of the SNP-string analysis are presented separately for the DRB1 and the MMEL1 loci.

### The DRB1 Cluster

#### Demographic data

For the DRB1 locus 18 SNP-strings were “identified” unambiguously in the population and another 3 were “identified” by the secondary analysis (Table 4). One further SNP-string was added to the “identified” list because its observed frequency was 2% following the “complete” analysis using all “possible” and “identified” SNP-strings (Table 2). Of these 22 “identified” SNP-strings, however, only (a1), (a2), (a3), (a4), (a6), and (a7) were present in sufficient numbers to have more than 1 homozygous individual (Figure 3). SNP-strings (a9) and (a13) were homozygous in 1 individual each. The “complete” SNP-string list included an additional 10 SNP-strings, although only two of these - (00000011111); or (a23) and (01010100000); or (a32) - were selected in more than a single individual. Moreover, each of these 10 additional SNP-strings on the “complete” list had a final estimated frequency, which rounded to zero.

**Table 4:** SNP-Strings “identified” by the SNP-String Analysis*.

DRB1 Locus			
Label	Unambiguous SNP-Strings	Label	Additional SNP-Strings
a1	00000000000	a19	10000010010
a2	01101100000	a20	10000010000
a3	10000100000	a21	10000011101
a4	01011000000	a22	00000011101
a5	01010000000		
a6	10000011111		
a7	10000011110		
a8	01111000000		
a9	10011100000		
a10	10001100000		
a11	01001000000		
a12	01011100000		
a13	01111001110		
a14	00000010000		
a15	01100100000		
a16	00000011110		
a17	01101000000		
a18	10000000000		
**MMEL1 Locus**			
**Label**	**Unambiguous SNP-Strings**	**Label**	**Additional SNP-Strings**
a1	01110110101	a17	00000101000
a2	01110010101	a18	01111010001
a3	00000000010	a19	01010000001
a4	11100001001	a20	00000001000
a5	01111010101	a21	11000000010
a6	00000101001	a22	00010110101
a7	00000001001	a23	01110000011
a8	00000000011	a24	11110010101
a9	11100001000		
a10	01110110100		
a11	01100001001		
a12	01111010100		
a13	11100000010		
a14	01010000000		
a15	01100000010		
a16	00100000010		

*Only “identified” SNP-strings are displayed in the Table (see text). Other “novel” SNP-strings, which had a frequency that rounded to 0 and are not included.

Of the 1857 participants in this study, 54 had missing data in the DRB1 region and, therefore, their subject-vectors could not be constructed. Of the remaining 1803 subjects, 652 (36%) had unambiguous SNP-string identifications. Also, using the final “complete” SNP-string list for the decomposition, 1,084 of the DRB1 SNP-strings identifications were non-conflicted, whereas 719 were conflicted. Of the conflicted identifications, however, only selected SNP-strings were involved in the conflict. Thus, for example, the (a2) SNP-string was involved in only 256 of the conflicts. All subject-vectors could be explained either as a combination of two of the 22 “identified” SNP-strings or as a combination of a “novel” SNP-string with one of the 22 “identified” SNP-strings. In no circumstance, was there a need to postulate two “novel” SNP-strings to explain any subject-vector. Thus, for the 3,606 haplotypes present in the study population, the set of “identified” SNP-strings set (see Table 4) was sufficient to explain 3,597 or 99.8% of the haplotypes, which are present in the population. In addition, this set was adequate to explain completely all but 20 (98.9%) of the subject-vectors. The “complete” set of SNP-strings was sufficient to explain 100% of the subject-vectors.

In addition, the SNP-strings found at the DRB1 locus seemed to be related evolutionarily. Thus, these SNP-strings could be divided into four apparent “families” (Table 5), which are related in the sense both that they each share certain structural features in common and that every family member can be derived by the change of a single SNP (0 to 1 or vice versa) from some other family member (i.e., each member had a Hamming distance of 1 from some other family member). For example, family #1 all had zeros for SNPs (n2–n6) and had ones for SNPs (n7–n9). Family #2 all had zeros for SNPs (n1 & n7–n11) and a one for SNP (n2). Family #3 all had zeros for SNPs (n2, n3, n8, n9 & n11). Family #4 consisted of a single member (a13), which could have resulted from a cross-over event between SNPs (n7 & n8) of SNP-strings (a7 & a8) to produce SNP-strings (a13 & a20). If so, this event occurred within the longest stretch of untagged DNA at this locus (Figure 1) and interconnects the DRB1 SNP-string families.

**Table 5:** Families of “identified” SNP-Strings.

DRB1 Locus			
Label	Family #1	Label	Family #3
a6	10000011111	a1	00000000000
a7	10000011110	a3	10000100000
a16	00000011110	a9	10011100000
a21	10000011101	a10	10001100000
a22	00000011101	a14	00000010000
		a18	10000000000
	**Family #2**	a19	10000010010
a2	01101100000	a20	10000010000
a4	01011000000		
a5	01010000000		**Family #4**
a8	01111000000	a13	01111001110
a11	01001000000		
a12	01011100000		
a15	01100100000		
a17	01101000000		
**MMEL1 Locus**			
**Label**	**Family #1**	**Label**	**Family #3**
a3	00000000010	a1	01110110101
a6	00000101001	a2	01110010101
a7	00000001001	a5	01111010101
a8	00000000011	a10	01110110100
a17	00000101000	a11	01100001001
a16	00100000010	a12	01111010100
a20	00000001000	a15	01100000010
a22	00010110101	a18	01111010001
		a23	01110000011
	**Family #2**		
a4	11100001001		**Family #4**
a9	11100001000	a14	01010000000
a13	11100000010	a19	01010000001
a21	11000000010		
a24	11110010101		

#### Genetic associations

The status of the major susceptibility allele (DRB1*1501) was known for the US-population. The OR of disease for having one-copy of this allele was 3.12 (p<0.0001) whereas the OR for having two-copies was 9.24 (Table 6). This is in keeping with the previously reported observation that both the control populations and the MS populations are in HWE with respect to the 1501 allele [9]. Thus, the weighting scheme for homozygous non-carriers, heterozygous carriers, and homozygous carriers of this allele, at least for the US population, is geometric (1, w, w^2^), as it must be for the cases to be in HWE [9], [38]. This type of weighting has also been referred to as co-dominant or allelic [38]. The frequency of the DRB1*1501 allele in controls was 10% (Table 2).

**Table 6:** Association of DRB1 SNPs with MS (all participants).

DRB1												
	SNPs											
	n1	n2	n3	n4	n5	n6	n7	n8	n9	n10	n11	HLA*
**Cases**												
0-copies	363	225	400	538	215	285	582	587	587	687	817	262
1-copy	476	487	465	380	486	482	342	345	345	224	148	189
2-copies	135	262	109	54	273	207	50	42	42	43	9	35
**Controls**												
0-copies	223	361	569	489	351	392	432	449	449	571	687	346
1-copy	453	405	275	329	403	386	370	356	356	222	174	80
2-copies	204	116	38	63	128	104	80	77	77	60	21	5
OR 1 allele	0.65	1.92	2.41	1.05	1.97	1.72	0.69	0.74	0.74	0.84	0.72	3.12
OR 2 alleles	0.41	3.62	4.08	0.78	3.48	2.74	0.46	0.42	0.42	0.60	0.36	9.24
X^2^ (1 df) 1 allele	16.7	37.2	78.0	0.2	38.5	27.5	14.7	9.3	9.3	2.5	7.4	55.5
X^2^ (1 df) 2 allele	42.3	72.3	37.9	1.0	71.2	39.8	10.0	11.7	11.7	3.9	3.6	19.0
HLA* Correlation	−0.47	0.58	0.78	−0.23	0.57	0.59	−0.31	−0.27	−0.27	−0.27	−0.13	na
Allelic Frequencies												
cases	0.38	0.52	0.35	0.25	0.53	0.46	0.23	0.22	0.22	0.16	0.09	0.27
controls	0.49	0.36	0.20	0.26	0.37	0.34	0.30	0.29	0.29	0.20	0.12	0.10

*HLA indicates the DRB1*1501 status. Data only available for US population.

Only SNPs (n2, n3, n5, & n6) were positively correlated with DRB1*1501 status (Table 6). All other correlations were negative (Table 6). The highest correlation observed was for (n3) and was (r = 0.78). The other positive correlations ranged from 0.57 to 0.59 (Table 6). The ORs for possessing 1 copy of these SNPs ranged from 1.72 to 2.41 whereas, for possessing 2 copies of each SNP, the ORs ranged from 2.74 to 4.08 (Table 6). All of these associations were highly significant although the observed ORs were substantially below those for the actual DRB1*1501 status, particularly for the homozygous association (see above).

By contrast, the SNP-string analysis provided a much closer correspondence to both MS status and DRB1*1501 status (Table 7). The OR (of disease) for possessing 1 copy of the (a2) SNP-string was 3.12 whereas for 2 copies of this SNP-string, the OR was 6.94 (p<0.0001). The allelic frequency of (a2) in controls was 12% (Table 3). The (a2) status was also highly correlated with DRB1*1501 status (r = 0.979) within the US-population where the DRB1 status was known (Table 9). As shown in Table 8, the OR observed in the US-population for possessing 2 copies of the (a2) SNP-string was substantially larger (OR = 8.95), in keeping with the known distribution of DRB1*1501 in this population. In Europe it was somewhat less (OR = 5.68) although the difference was not statistically significant and, in the European population, the actual DRB1*1501 distribution is not known. By contrast, the disease risks for heterozygotes from Europe (OR = 3.20) and from the US (OR = 3.05) were quite similar between regions, as were the allelic frequencies in both cases and controls (Table 3).

**Table 7:** Association of DRB1 SNP-Strings with MS (all participants)*.

DRB1							
	SNP-Strings						
	a1	a2	a3	a4	a7	a8	a9
**Cases**							
0-copies	810	483	677	695	735	848	917
1-copy	138	404	250	235	209	101	36
2-copies	5	66	26	23	9	4	0
**Controls**							
0-copies	645	660	549	616	601	733	817
1-copy	197	177	261	219	226	111	32
2-copies	8	13	40	15	22	6	1
OR 1 allele	0.56	3.12	0.78	0.95	0.76	0.79	1.00
OR 2 alleles	0.50	6.94	0.53	1.36	0.34	0.58	0
X^2^ (1 df) 1 allele	22.9	114.7	5.7	0.2	6.3	2.7	0
X^2^ (1 df) 2 allele	1.5	50.9	6.3	0.8	8.2	0.7	1.1

*Data is taken from the “complete” analysis (see text). Only selected SNP-strings are displayed.

**Table 8:** Association of DRB1 SNP-Strings with MS (US particcipants)*.

DRB1							
	SNP-Strings						
	a1	a2	a3	a4	a7	a8	a9
**Cases**							
0-copies	405	250	332	357	361	417	453
1-copy	64	188	125	106	105	53	19
2-copies	3	34	15	9	6	2	0
**Controls**							
0-copies	319	329	272	323	278	353	393
1-copy	93	81	125	87	123	60	22
2-copies	3	5	18	5	14	2	0
OR 1 allele	0.54	3.05	0.82	1.10	0.66	0.75	0.75
OR 2 alleles	0.79	8.95	0.68	1.63	0.33	0.85	na**
X^2^ (1 df) 1 allele	11.9	52.5	1.8	0.4	7.4	2.1	0.8
X^2^ (1 df) 2 allele	0.1	28.2	1.1	0.8	5.5	0	na**

*Data is taken from the “complete” analysis (see text). Only selected SNP-strings are displayed.

**na  =  not available (has a zero divisor).

**Table 9:** Contingencies for HLA DRB1* Status and (a2)-haplotype Status from SNP-String Phasing Method*.

SNP-string (a2) Status	HLA DRB1*1501 Status		
	0-Copies	1-Copy	2-Copies
**0-Copies**	573	6	0
**1-Copy**	5	264	0
**2-Copies**	0	0	39

Correlation of (a2)-haplotype status with HLA DRB1*1501 = 0.981.

*Including the change in status for the one subject who was re-typed for DRB1*1501 and found to be heterozygous instead of homozygous (see text).

In addition, from Table 7, it appears that SNP-strings (a1, a3, a7, & a8) may be protective. Nevertheless, this is an illusion. When the (a2) SNP-string is removed from the analysis, the apparent protective effect vanishes. Thus, the protective effect of these SNP-strings lies in the fact that carriers are less likely to also carry the (a2) SNP-string.

Closer examination of the 12 individuals who accounted for the non-perfect correlation of (a2) status with DRB1*1501 status revealed that only five (a2) SNP-string carriers (an a2–a7; an a2–a27; and three a2–a3 heterozygotes) did not also carry the 1501 allele. In addition, one individual (an a2–a21 heterozygote) was homozygous for the 1501 allele, which implies that the (a21) SNP-string can also carry this allele. However, the (a21) SNP-string is very different from the (a2) SNP-string (Table 5). The (a21) SNP-string is from a separate family and differs from (a2) in 9 out of the 11 SNP-positions (Table 5). It is, therefore, hard to rationalize (short of invoking some double crossover event or exon-exchange) whereby this linkage would be possible. Moreover, for every other (a21)-carrier, this SNP-string either didn’t harbor the 1501 allele or the individual was both an (a2–a21) and a DRB1*1501 heterozygote so that, other than in this one instance, it was never necessary to posit that an (a21) SNP-string harbored the 1501 allele. It seemed plausible, therefore, that an error had been made in the typing of this subject’s DRB1 status. On this basis, the typing for this individual was rechecked and, on repeat typing this individual was found to be a (1501/0701) heterozygote. This change slightly improved the correlation between (a2)-status and HLA DRB1 status (r = 0.981). The remaining 6 subjects were DRB1*1501 heterozygotes, who lacked the (a2) SNP-string but included (in part) SNP-strings (a3) or (a10), which seem more plausible as 1501 carriers from an evolutionary perspective (Table 5).

### The MMEl1 Cluster

#### Demographic data

For the MMEL1 locus 16 SNP-strings were identified unambiguously in the population and another 8 by the secondary analysis (Table 4). No additional “identified” SNP-strings were found following the “complete” analysis using all “possible” and “identified” SNP-strings. Of these 24 “identified” SNP-strings, however, only (a1), (a3), (a4), (a5), (a7), and (a14) were present in sufficient numbers to have more than 1 homozygous individual (Figure 3). SNP-strings (a6), (a13), and (a15) were homozygous in 1 individual each. Moreover, in the case of this locus, the (a3) SNP-string was responsible for about 45% of the identifications in both cases and controls (Figure 3). In the “complete” SNP-string list, there were an additional 17 SNP-strings identified although only 4 of these (00000110101, 00100001001, 00000000101, 00100000001) occurred in more than two individuals. All of these “novel” SNP-strings on the “complete” list had a final estimated frequency, which rounded to zero.

Of the 1,857 participants in this study, 10 had missing data in the MMEL1 region and, therefore, the MMEL1 subject-vectors could not be constructed. Of the remaining 1,847 participants, 536 (29%) had unambiguous SNP-string identifications. Also, using the final “complete” SNP-string list for the decomposition, 845 of the SNP-string identifications were non-conflicted whereas 1,002 were conflicted. Again, only selected SNP-strings were involved in each conflict. For example, the (a4) SNP-string was involved in only 271 of the conflicts. All subject-vectors could be explained either as a combination of two of the 24 “identified” SNP-strings or as a combination of a “novel” SNP-string with one of the 24 “identified” SNP-strings. In no circumstance, was there a need to postulate two “novel” SNP-strings to explain any subject-vector. Thus, for the 3,694 haplotypes present in the study population, the set of “identified” SNP-strings set (see Table 4) was sufficient to explain 3,660 or 99.1% of the haplotypes, which are present in the population. In addition, this set was adequate to explain completely all but 34 (98.2%) of the subject-vectors. Again, the “complete” set of SNP-strings was sufficient to explain 100% of the subject-vectors.

As was the case for the DRB1 locus, the MMEL1 locus also seemed to consist of related families of SNP-strings although, in this region, the families are more interconnected and, thus, the distinction of one family from another was less clear-cut (Table 5). Nevertheless, family #1 all had zeros for SNPs (n1, n2, & n5). Family #2 all had ones for SNPs (n1 & n2) and zeros for SNPs (n4 & n5). Family #3 all had zeros for SNPs (n1) and ones for SNPs (n2 & n3). Family #4 only had two members, which differed from each other only at SNP (n11).

#### Genetic associations

The genetic association for each of the 11 SNPs in the MMEL1 region are presented in Table 10. None of the ORs for either 1 or 2 copies of any SNP in this region were more than marginally significant (Table 10). Nevertheless, using SNP-strings, there was significant association between the (a4) SNP-string and MS, with the OR for possessing 2 copies of this allele being 2.93 (Table 11). Moreover, this was replicated in both independent subpopulations with the OR in Europe being 2.86 and the OR in the US being 2.96. By contrast, the OR for possessing 1 copy of this allele was 0.90. Presumably, therefore, this susceptibility allele acts in an autosomal recessive manner. The allelic frequency of (a4) in the control population was 11% (Table 2).

**Table 10:** Association of MMEL1 SNPs with MS (all participants).

MMEL1											
	SNPs										
	n1	n2	n3	n4	n5	n6	n7	n8	n9	n10	n11
**Cases**											
0-copies	717	289	304	473	721	694	494	631	495	259	258
1-copy	222	476	477	412	230	256	401	281	400	476	480
2-copies	35	209	193	89	19	24	79	62	79	239	235
**Controls**											
0-copies	654	251	268	403	661	601	428	547	430	252	199
1-copy	204	418	421	380	199	259	373	297	372	447	465
2-copies	22	213	193	98	20	22	81	38	80	183	217
OR 1 allele	0.99	0.99	1.00	0.92	1.06	0.86	0.93	0.82	0.93	1.04	0.80
OR 2 alleles	1.45	0.85	0.88	0.77	0.87	0.94	0.85	1.41	0.86	1.27	0.84
X^2^ (1 df) 1 allele	0.0	0.0	0.0	0.7	0.3	2.2	0.5	3.8	0.5	0.1	4.0
X^2^ (1 df) 2 allele	1.8	1.3	0.8	1.7	0.1	0.0	0.6	1.5	0.5	2.8	1.6
Allelic Frequencies											
cases	0.15	0.46	0.44	0.30	0.14	0.16	0.29	0.21	0.29	0.49	0.49
controls	0.14	0.48	0.46	0.33	0.14	0.17	0.30	0.21	0.30	0.46	0.51

**Table 11:** Association of MMEL1 SNP-Strings with MS (all participants).

MMEL1							
	SNP-Strings						
	a1	a3	a4	a5	a6	a7	a14
**Cases**							
0-copies	734	288	762	732	935	881	955
1-copy	219	470	182	219	35	84	14
2-copies	17	212	26	19	0	5	1
**Controls**							
0-copies	637	289	686	668	845	792	859
1-copy	226	425	183	191	31	83	17
2-copies	14	163	8	18	1	2	1
OR 1 allele	0.84	1.11	0.90	1.05	1.02	0.91	0.74
OR 2 alleles	1.05	1.31	2.93	0.96	0	2.25	0.90
X^2^ (1 df) 1 allele	2.5	0.9	0.9	0.2	0	0.3	0.7
X^2^ (1 df) 2 allele	0	4.0	7.6	0	1.1	1.0	0

*Data is taken from the “complete” analysis (see text). Only selected SNP-strings are displayed.

### Comparisons with the SHAPEIT-2 Phasing Method

Running SHAPEIT-2 on the same 11-SNP data used by the SNP-string method and excluding subject-vectors that had missing data, the two methods were only 61% concordant in the DRB1 region and 75% concordant in the MMEL1 region. When the two methods predicted different haplotype combinations to explain a particular subject-vector, in general, the combinations chosen by SHAPEIT-2 had substantially lower likelihoods compared to those predicted by the SNP-string method. Indeed, this was even the case when estimating the haplotype frequencies based the SHAPEIT-2 predictions. In addition, to account for all of the subject-vectors, SHAPEIT-2 predicted the presence of many more “novel” haplotypes than the SNP-string method, despite the fact that the smaller set of haplotypes was sufficient to explain 100% of the subject-vectors. Thus, in the DRB1 region the SNP-string method invoked only 32 haplotypes, compared to 62 haplotypes using SHAPEIT-2, in order to explain 100% of the genotype (subject-vector) data. Similarly, in the MMEL1 region, SNP-string method invoked only 41 haplotypes, whereas SHAPEIT-2 invoked 57 haplotypes. In both regions, the additional haplotypes identified by SHAPEIT-2 were never unambiguously present in the subject-vector data. Finally, although a highly significant association of the (a2) haplotype with HLA DRB1*1501 status (r = 0.760) was found using the SHAPEIT-2 method, the strength of this association was significantly less (p<0.0001) compared to that found using the SNP-string method (r = 0.981).

By contrast, when the length of the SNP data included in the SHAPEIT-2 analysis was increased ten fold (to a span of 2,000 kb surrounding the 11-SNP sequences used in the above analyses) the results of the 2 methods were almost identical. Thus, the two methods were concordant in 99.4% of individuals in the DRB1 region and 99.1% of individuals in the MMEL1 region. Many of the discrepancies occurred because the one method included haplotypes, which were not on the other method’s list. Also, compared to the SNP-string method, SHAPEIT-2 predicted an additional 2 haplotypes in the DRB1 region and an additional 3 haplotypes in the MMEL1 region. In most of these case there is no way to determine which method is more accurate. In the DRB1 region, for two such individuals, however, there was additional information. In both individuals, the SNP-string method predicted the (a2) allele whereas SHAPEIT-2 did not. Only one of these individuals carried the DRB1*1501 allele. Consequently, provided these individuals were typed correctly, and assuming that DRB1 status is a good surrogate for (a2) status, each method was correct only once. For the remaining discrepancies in the DRB1 region, the two methods chose combinations from the same subset of SNP-strings so that probabilistic comparisons were possible. Using the haplotype frequencies estimated from the SHAPEIT-2 output, the SNP-string method predicted combinations were more probable than the SHAPEIT-2 predictions in every case. In these circumstances, the ratios of the two probabilities ranged from 1.05 to 619 in favor of the SNP-string method.

**Figure 4:**
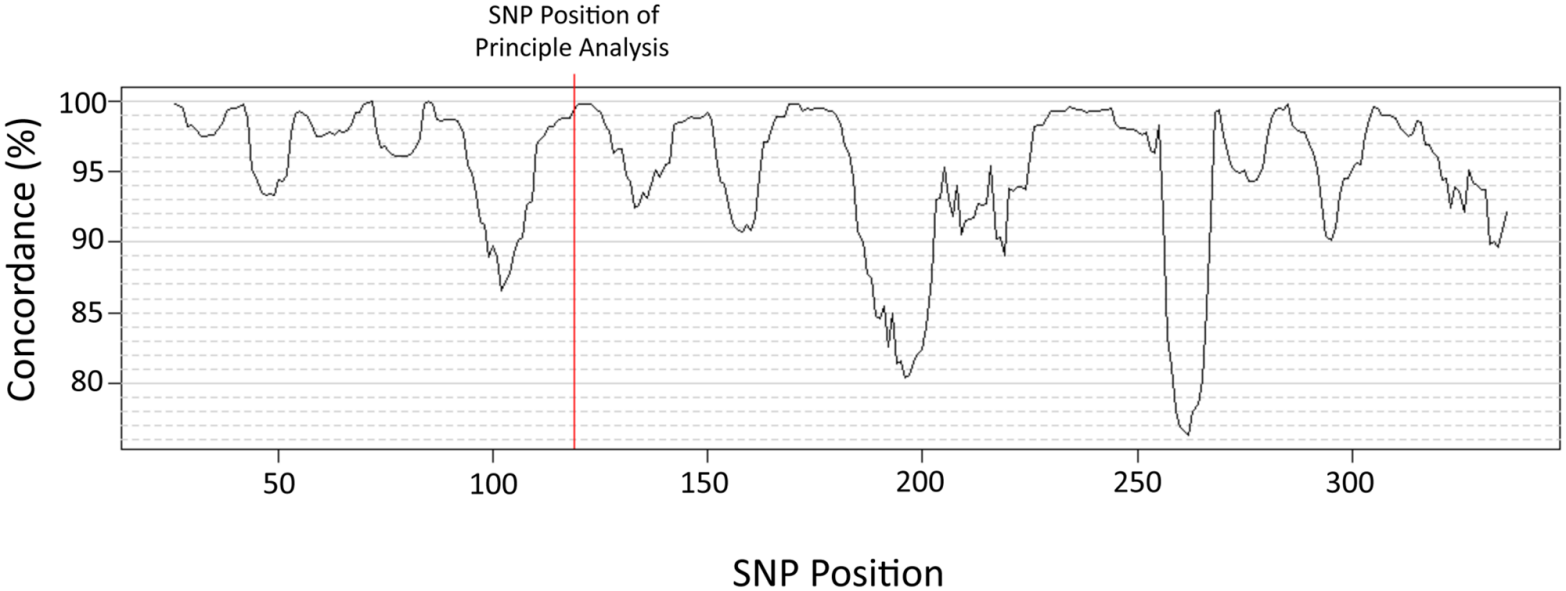
Concordance of the two SHAPEIT-2 analyses depending upon the SNP- position of the initial SNP used for the haplotype analysis. The first SHAPEIT-2 analysis predicted the complete haplotypes (across the entire region) starting at SNP-position 1. The second SHAPEIT-2 analysis predicted the complete haplotypes starting at SNP-position 25. The 11-tuple subject-vectors for the principle analysis undertaken here began at SNP-position 119 (rs2234167), as indicated by the verticle red line.

Nevertheless, such high agreement between the two methods was not uniform throughout the 2,000 kb span of DNA but, rather, was characterized by peaks and valleys of agreement alternating throughout the span. For example, as noted above, in the MMEL1 region, when the 11-SNP sequence of subject vectors was started at SNP-position 119 (rs2234167) the agreement between the two methods was 99.1%. By contrast, when the 11-SNP sequence was begun at SNP-position 100 (rs1129333) the agreement between the two methods fell to 66.6%. Moreover, in this region there was, again, a marked disparity between the two methods in the number of haplotypes needed to explain 100% of the subject-vectors. Thus, in this region, the SNP-string method predicted only 120 haplotypes, whereas SHAPEIT-2 predicted the presence of 173 haplotypes (i.e., using either method, the variability of haplotypes in this region was 3–4 times the variability found in a DNA region only 150 kb away). Because the SNP-string method is based upon only the local 11-tuple subject-vectors, the outcome of this method depends only upon the (known) identity of the subject-vectors in the population. By contrast, because SHAPEIT-2 seemed to perform poorly when the input was limited to the 11-tuple subject-vectors of interest, it also seemed possible that SHAPEIT-2 might perform less well depending upon where (in the genome) the haplotype analysis was begun. Consequently, we ran the SHAPEIT-2 program starting at two different genomic locations. The first SHAPEIT-2 analysis began at SNP-position 1 (rs2031709); the second began at position 25 (rs6603813). The concordance of the predictions from these two SHAPEIT-2 analyses were then compared for each 11-tuple across the region (Figure 4). As can be seen from the figure, the concordance between the two SHAPEIT-2 analyses is very similar to the concordance between the two haplotype methods, being characterized by peaks and valleys of agreement alternating throughout the span of DNA (Figure 4).

## Discussion

Currently, many groups throughout the world conduct GWAS studies to identify genomic regions that are associated with complex human diseases [14], [15], [17]–[21]. Typically, gene chips are used to interrogate ∼1,000,000 known human SNPs genome-wide in a sample population. If a SNP or several SNPs in a particular region are associated with the disease then it is presumed that some allele of a nearby gene is responsible for the observed association, thus GWAS are designed to identify genomic regions of association. The difficulty with this approach, however, is that the associations are often weak and require thousands of patients to uncover [14], [15]. Moreover, at least for the DRB1 locus, each associated SNP has a much greater allelic frequency compared to the underlying susceptibility allele, which is an example of synthetic association [39]. Thus, the allelic frequency for the DRB1*1501 allele in the control population was 10%, whereas for the four most associated SNPs it ranges from 20% to 37% (Table 7). Also, unless the SNP is itself produces the associated genetic abnormality, it may difficult to determine which allele is responsible for the association. Finally, some probabilistic approaches to haplotype identification [25]–[28] depend upon correlations with the disease and don’t really lead to identifications of the specific haplotypes, which exist at a given genetic location. Rather, these methods search the data for clusters of SNPs, which are (jointly) associated with the disease and, which, therefore, presumably belong to a particular disease-associated haplotype. They don’t actually define the haplotype. For example, knowing that the four SNPs (n2, n3, n5 and n6) tag a haplotype, in this case the (a2) SNP-string, which is associated with MS, is not the same as identifying the (a2) haplotype itself and doesn’t permit either testing of other haplotypic associations or comparing the genetic makeup of different populations (Table 3). Presumably, many of these potential difficulties could be mitigated if the two haplotypes at a given genetic locus could be identified confidently for each individual.

There is a high degree of confidence in the identity of those predicted haplotype combinations, which include “identified” SNP-strings. Thus, the large majority of these “identified” SNP-strings are present unambiguously and, for the others, their presence is implicated repeatedly in many different individuals. The degree confidence in the identity of those combinations that include “novel” SNP-strings is more tentative because, in most cases, these are present in only a few individuals, they are never identified uniquely, and, for the purposes of their selection, they are “assigned” a uniform (arbitrarily low) frequency. Nevertheless, because the vast majority of persons (>98%) have a combination, which includes 2 “identified” SNP-strings, the phasing method proposed here identifies, with a high degree of confidence (for the vast majority of the population), the 2 SNP-haplotypes, which make up a person’s genotype. Thus, the SNP-string method mitigates many of the potential problems discussed earlier. First, in regions spanning approximately 200 kb of DNA, only a limited number of SNP-strings (SNP-haplotypes) seem to exist within the case-control populations from Europe and the US (Figure 3; Table 3). Indeed, as anticipated because both populations were largely of northern European origin, the frequency distribution of the different SNP-strings was almost identical in the two groups (Table 3). Moreover, the identification of the constituent SNP-string haplotype for each genotype are, for the most part, either unambiguous or the unique combination of two “identified” SNP-strings. Consequently, there is little doubt that these SNP-strings (so identified) represent the actual haplotypes, which cover the entire 200 kb segment and, consequently, this method should facilitate comparisons regarding the genetic make-up different human populations in specific genetic regions.

Although the analysis presented here represents only two loci, the same pattern pertained to every locus screened preliminarily (including MS-associated intergenic genomic regions). Naturally, the “identified” SNP-strings, likely represent sub-families of related haplotypes, which have not been separately defined. For example, only 22 SNP-strings were “identified” at the DRB1 locus (Table 4), whereas there are known to be hundreds of DRB1 alleles [40], [41]. Some of these alleles are either very rare or only present in non-European ethnic groups [40], [41] and might not be present in our sample. Others, however, likely, share the same 11-tuple SNP-string. Including a larger number of SNPs in the SNP-string would permit finer distinctions to be made between alleles. However, this will also probably decrease the number of unambiguous SNP-string identifications. Similarly, reducing the length of DNA covered by an 11-tuple SNP-string will also increase the allelic separation but may also reduce the number of unambiguous identifications and the number of “identified” SNP-strings. Each of these changes could be either good or bad, depending on which effects predominate. Unquestionably, therefore, each of these variables will need to be studied systematically in order to determine the optimum number of SNPs and the optimum length of DNA to be included in the analysis.

Second, this SNP-string phasing method seems to overcome many of the objections to Clark’s original algorithm [25]–[27]. All “identified” SNP-strings are either “unambiguous” or they are unique pairings involving a “novel” with previously “identified” SNP-string. In addition, each of the SNP-string lists here developed (i.e., unambiguous “identified”, “identified”, and “complete”) and the associated haplotype frequencies are derived from repeated deconstructions of the entire dataset. Therefore, these lists (and the estimated haplotype frequencies) are independent of the order of data entry. Although the method does not guarantee the presence of “unambiguous” SNP-string identifications in the data, nevertheless, at every locus examined either in detail or preliminarily (including intergenic genomic regions), more than 16% of individuals (oftentimes much more) had “unambiguous” identifications, even when the SNP-string length was increased from 11 to 24. Also, these unambiguous identifications, when they are made, are independent of any distribution effects.

Although the SNP-string method does not directly assume that the population is in HWE, it does resolve halpotype conflicts based on the observed frequencies of the different non-conflicted SNP-strings in the population. Therefore, the method does imply the random combination of SNP-strings. Nevertheless, at least in the case of the DRB1 locus, the susceptibility allele is known to be at HWE [9]. Moreover, the fact that the frequency distribution of the different haplotypes is essentially identical in the European and US populations (Tables 2 and 3) suggests that each population is in a similar equilibrium state. However, regardless of the exact distribution, the combination of two rare “novel” SNP-strings is still anticipated to be less common than the combination of either two “identified” or one “novel” plus one “identified” SNP-string. Thus, only one subject-vector (out of approximately 25,000 screened preliminarily) could not be explained such a combination, which included, at least, one “identified” SNP-string. This suggests that the combination of two “novel” SNP strings is extremely rare, as anticipated if the population were composed of random SNP-string combinations. Also, the list of “identified” SNP-strings, generated by this method, is sufficient to account for more than 99% of the SNP-strings present in the population. Thus, the “identified” list may be short but it is also, largely, complete. Naturally, the “complete” SNP-string list accounts for 100% of the subject-vectors, although most of these additional SNP-strings were found in only a single individual. Because the actual phased haplotype information is not available, it is not possible to test the method directly for errors. Nevertheless, most of the identifications were either non-conflicted or, when conflicted, had one particular SNP-string pair, which was far more likely than the others. In addition, it is noteworthy that the haplotypes predicted by the SNP-string method in the DNA region studied here were virtually identical to those predicted by the SHAPEIT-2 algorithm when the span of DNA included in this latter analysis was increased 10-fold. This concordance of independent analysis methods, adds strong support for the notion that the haplotypes have been correctly identified in the vast majority of subjects. However, the agreement of the SNP-string method with SHAPEIT-2 was considerably less in other regions within the 2,000 kb span (Figure 4). Notably, the regions of discordance between algorithms occurred in regions where SHAPEIT-2 was also substantially discordant with itself (Figure 4). These regions also had especially high SNP-string variability and it is possible that the SHAPEIT-2 algorithm has difficulty in such circumstances.

Third, the identification of SNP-strings could be expanded to provide haplotype information over larger genomic segments. For example, suppose that the “identified” SNP-string combinations from two adjacent 11-tuple SNP-string combinations were known. Thus, suppose that the first combination was (01101100000) plus (01011010000); and the second was (01010000000) plus (10000011111). Suppose, further, that the combination of the overlapping 11-tuple segment, beginning at position 6 of the first 11-tuple, was (10000001010) plus (01000010000). In this case, the only possible 22 SNP-string haplotype configuration is (0110110000010000001010) plus (0101101000010000011111). Using similar (but expanded) logic, such an approach can be extended to provide haplotype information over increasingly large segments of the genome. Naturally, there is a need to optimize the number of SNPs and the length of DNA to be included in each SNP-string analysis and, no doubt, the method will require other refinements. Nevertheless, the haplotype information yielded by such a method would be reproducible and largely accurate over protracted regions of DNA. Naturally, in the future, next generation sequencing techniques (e.g., full exome sequencing) might replace some (or many) of the current phasing methods. However, even if these methods becomes readily available and can overcome their own phasing issues, specific methods might fail to identify disease-associations with intergenic regions, some of which have already been found in the GWAS published by the International Consortium [14], [15].

Fourth, and most importantly, this method permits considerably more powerful tests of genetic association. The advantage of this method over the simple SNP-analysis is underscored by two examples. First within this dataset, there was no SNP-association of the MMEL1 locus with the MS disease, even for the SNP that was identified in the much larger GWAS as being highly MS-associated [14]. Moreover, although highly significant, this association only had an OR of 1.16 [14]. By contrast, the SNP-string method, using the same SNP data, was able to detect a significant and replicable association of this locus with MS and the OR was considerably higher (2.93). Second, at the DRB1 locus, although there were highly associated SNPs, the highest correlation with actual DRB1*1501 status was for the (n3) SNP-string, only modest (<0.78), and had an OR of only 4.08 for the homozygous association (compared to more than double that for the actual allele). Moreover, this SNP had a frequency twice that of the DRB1*1501 allele, so that even for this SNP, less than half of the alleles tagged were the correct one. By contrast, using the SNP-string method, the (a2) SNP-string had a correlation with the DRB1*1501 allele of 0.981, had a frequency comparable to that of DRB1*1501, and had an OR also more than double that observed for the (n3) SNP.
